# Propuesta de un modelo conceptual para el estudio de los ambientes alimentarios en Chile

**DOI:** 10.26633/RPSP.2017.169

**Published:** 2017-12-05

**Authors:** Patricia Gálvez Espinoza, Daniel Egaña, Dominique Masferrer, Ricardo Cerda

**Affiliations:** 1 Departamento de Nutrición Facultad de Medicina, Universidad de Chile Chile Departamento de Nutrición, Facultad de Medicina, Universidad de Chile, Chile.; 2 Departamento de Atención Primaria y Salud Familiar Facultad de Medicina, Universidad de Chile Chile Departamento de Atención Primaria y Salud Familiar, Facultad de Medicina, Universidad de Chile, Chile.; 3 Escuela de Salud Pública Universidad de Chile Chile Escuela de Salud Pública, Universidad de Chile, Chile.

**Keywords:** Ambiente, conducta alimentaria, determinantes sociales de la salud, Chile, Environment, feeding behavior, social determinants of health, Chile, Meio ambiente, comportamento alimentar, determinantes sociais da saúde, Chile

## Abstract

**Objetivo.:**

Describir una propuesta de modelo conceptual para sistematizar los factores que condicionan los ambientes alimentarios y la forma en que estos se expresan en la conducta alimentaria de la población chilena.

**Métodos.:**

Estudio realizado en Chile que incluyó cuatro etapas secuenciales e iterativas: revisión de la literatura, apertura de discusión del modelo preliminar, generación de un segundo modelo y nueva discusión con expertos y elaboración de modelo conceptual definitivo.

**Resultados.:**

Se elaboró un modelo conceptual que incluye cinco ambientes alimentarios interrelacionados, aunque con características distintivas: ambiente doméstico, ambiente vía pública, ambiente institucional y organizacional, ambiente de restauración y ambiente de abastecimiento. Se sitúan al sistema alimentario y a la cultura como las variables más amplias del modelo. Los determinantes sociales de los ambientes alimentarios y alimentación constituyen los factores estructurales e intermedios de estos ambientes. En una relación más directa con los ambientes, se hallan la industria alimentaria y las políticas en alimentación y nutrición. Por último, se incluye al individuo y la cohesión social como parte del modelo, ya que son los individuos o los grupos quienes transitan dentro de los ambientes alimentarios.

**Conclusiones.:**

Este modelo representa un conjunto de definiciones, conceptos y relaciones que tienen una interacción compleja y multidireccional, por lo que el modelo contribuye a comprender de forma integral el fenómeno de cómo son condicionados los ambientes alimentarios sobre la conducta alimentaria.

## SUMMARY

**Objective.:**

Describe a proposal for a conceptual model to systematize the factors affecting food environments and the ways in which these factors are expressed in food behaviors in the Chilean population.

**Methods.:**

Study conducted in Chile, with four sequential and iterative stages: literature review, initial discussion of the preliminary model, development of a second model and further discussion with experts, and development of a definitive conceptual model.

**Results.:**

A conceptual model was developed, including five interrelated food environments with distinctive characteristics: domestic environment, street environment, institutional and organizational environment, restaurant environment, and food supply environment. The food system and food culture are considered the broadest variables in the model. The social determinants of the food environments and eating habits constitute structural and intermediate factors in these environments. The food industry as well as food and nutrition policies are more directly related to food environments. Finally, individuals and social cohesiveness are included in the model, since individuals and groups move within food environments. *Conclusions*. This model presents a set of definitions, concepts and relationships with complex and multidirectional interactions. The model, therefore, contributes to a comprehensive understanding of how food environments condition food behavior.

## RESUMO

**Objetivo.:**

Propor um modelo conceitual para sistematizar os fatores que condicionam os ambientes alimentares e como eles são expressos no comportamento alimentar da população chilena.

**Métodos.:**

Estudo realizado no Chile que compreendeu quatro etapas sequenciais e iterativas: revisão da literatura, abertura de discussão do modelo preliminar, geração de um segundo modelo e nova discussão com especialistas e elaboração do modelo conceitual definitivo.

**Resultados.:**

Foi elaborado um modelo conceitual que engloba cinco ambientes alimentares inter-relacionados, apesar de terem características distintas: ambiente doméstico, ambiente público, ambiente institucional e organizacional, ambiente de restauração e ambiente de abastecimento. O sistema alimentar e fatores culturais são as variáveis mais amplas do modelo. Os determinantes sociais dos ambientes alimentares e da alimentação constituem fatores estruturais e intermediários destes ambientes. A indústria alimentícia e políticas de alimentação e nutrição têm uma relação mais direta com os ambientes. Por último, são incluídos no modelo os indivíduos e a coesão social, visto que são os próprios indivíduos ou grupos de indivíduos que circulam dentro dos ambientes alimentares.

**Conclusões.:**

Este modelo representa um conjunto de definições, conceitos e relações com interações complexas e multidirecionais. Ele contribui para compreender como um todo o fenômeno de como os ambientes alimentares estão condicionados ao comportamento alimentar.

Existe consenso acerca de que los enfoques de intervención individual no son suficientes para mejorar conductas de la población (1). Así, la noción de ambiente alimentario (AA) ha ganado espacio en la literatura que estudia la conducta alimentaria (CA), al constituir un factor fundamental que interviene, mediante la facilitación o la obstaculización, la elección y el consumo de alimentos (2–4). De esta forma, la conducta de un individuo solo puede orientarse a realizar elecciones alimentarias saludables si cuenta con un ambiente con disponibilidad y acceso a los alimentos (2, 5).

Entonces, estudiar los AA se vuelve imperante, para comprender cómo las conductas individuales son condicionadas por contextos mayores y para orientar intervenciones nutricionales colectivas implementadas en el nivel local, regional o nacional. De esta manera, la elaboración de modelos conceptuales integradores, con pertinencia nacional, permite comprenderla interrelación de los distintos factores que condicionan los AA. Esto contribuyó, por ejemplo, a aumentar la efectividad de las intervenciones realizadas para frenar el aumento de la prevalencia de obesidad (6). En Chile, se ha establecido hace poco tiempo la Ley 20 606 que regula la composición y publicidad de alimentos (7). Se ha comenzado también a desarrollar un programa de vigilancia y fiscalización de AA (8), con la generación de índices de carácter internacional (9, 10). En este contexto, se requiere debatir qué indicadores tendrán mayor pertinencia local y que permitan monitorear políticas públicas implementadas.

El presente trabajo describe una propuesta de modelo conceptual (MC) para sistematizar los factores que condicionan los AA y la forma en que estos se expresan en la CA de la población chilena.

## MATERIALES Y MÉTODOS

Estudio de tipo exploratorio con análisis conceptual (11, 12). La metodología fue elaborada en común acuerdo con el Departamento de Nutrición y Alimentos del Ministerio de Salud de Chile (MINSAL) y puede ser revisada en detalle en el reporte elaborado (13).

### Revisión literaria

**FIGURA 1.**
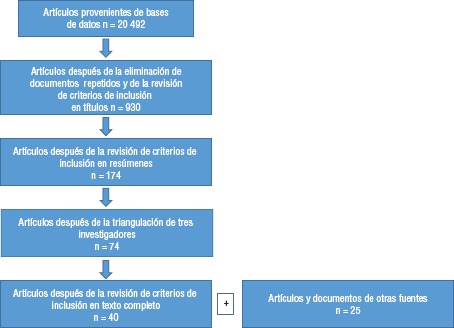
Flujograma de revisión bibliográfica

**FIGURA 2.**
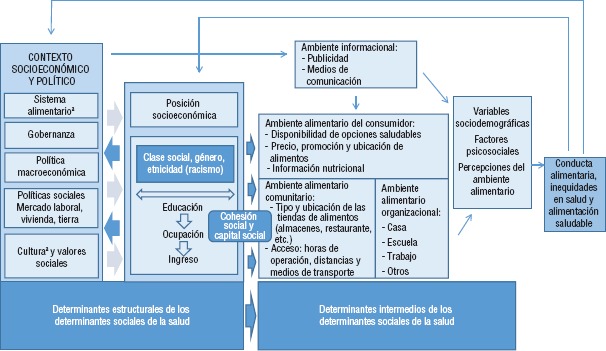
Modelo conceptual prelimitar diseñado a partir de la revisión de la literatura

^a^Sistema alimentario y cultura poseen expresiones en niveles intermedios, colectivos e individuales del modelo.

Se realizó una búsqueda bibliográfica de artículos relacionados con el tema de AA en base de datos generales como EBSCO y Scopus, y bases especializadas como MEDLINE, Web of Science, entre otras (figura 1). Las palabras clave utilizadas (en inglés para lograr un mayor alcance) fueron “Eating or food or feeding or behavior or conduct or action or habits or diet + Environmental factor or economic factor or availability factor or store factor or variable or condition or level or dimension”. Los criterios de inclusión fueron: (1) artículos científicos con revisión de pares publicados entre el 2010 y 2015; (2) se incluyeron revisiones sistemáticas, estudios aleatorios controlados, estudios preintervencióny posintervención, cuasi experimentales, estudios transversales y otros estudios no experimentales; (3) los artículos debieron contener tanto factores ambientales como variables de análisis, ya sea de forma exploratoria o en intervenciones; y (4) los artículos debían permitir definir factores, determinantes o variables en base a la descripción y análisis que realizan los investigadores. Se excluyeron: (1) intervenciones en ambientes experimentales (por ej., laboratorios); (2) artículos referidos a la actividad física; (3) estudios en animales; (4) artículos referidos a los cambios en variables individuales; y (5) estudios referidos a la prevención de otras enfermedades relacionadas con la nutrición sin relación con la obesidad o enfermedades crónicas no transmisibles (por ej., caries dentales). Los artículos seleccionados se estudiaron mediante análisis temático, en el que se distinguían aspectos como definiciones, efectos y condicionantes de AA. Luego se reorganizaron los resultados con organizadores de la información el modelo ecológico definido por Glanz y cols. sobre prácticas alimentarias (14), el modelo de determinantes sociales de la salud (DSS) (15) y el modelo del sistema alimentario de Goody y cols. (16), los cuales permitieron desarrollar el modelo conceptual preliminar (MCP), que incluyó cuatro AA y sus condicionantes (figura 2).

### Apertura de discusión del modelo preliminar

Con el MPC desarrollado, se realizó un seminario con expertos nacionales en el área de alimentación y nutrición, entre los que se encontraron profesionales de, por ejemplo, la Agencia Chilena para la Inocuidad Alimentaria, la Junta Nacional de Jardines Infantiles, la Organización de las Naciones Unidas para la Agricultura y la Alimentación (FAO, por sus siglas en inglés) y la Organización Panamericana de la Salud (OPS), entre otros. El propósito fue evaluar el MCP en relación a conceptos y factores condicionantes. Se convocó a 55 expertos, de los cuales asistieron 31. La metodología del seminario incluyó la presentación del proceso realizado para la elaboración del MCP, para luego armar grupos de discusión en torno a este. Los resultados de la discusión de los grupos fueron sistematizados en un segundo MCP.

Además, se establecieron tres instancias de diálogo con la ciudadanía, en Antofagasta (zona Norte), Santiago (zona Centro) y Concepción (zona Sur). Para cada encuentro, se convocó a personas de distintas organizaciones sociales y de la sociedad civil vinculadas a la salud y la alimentación. Esta instancia tuvo el mismo propósito del seminario. Cada encuentro siguió la metodología propuesta por MINSAL(17), que incluye una etapa informativa, una deliberativa, y una integrativa.

Adaptado a partir del modelo ecológico definido por Glanz y cols. sobre prácticas alimentarias (14), el modelo de determinantes sociales de la salud (DSS) (15), y el modelo del sistema alimentario de Goody y cols. (16)

Al encuentro realizado en el Norte del país asistieron 14 participantes, 11 al encuentro en Santiago (de 19 convocados) y 14 al que se organizó en el Sur. La convocatoria en el Norte y en el Sur de país fue encomendada a las secretarías regionales ministeriales (SEREMI)de salud de Antofagasta y del Biobío, que no reportaron el número de convocados.

### Generación de un segundo MC y nueva discusión con expertos

Los resultados de las discusiones realizadas fueron sistematizados en un segundo MC, en el que se incluyeron la crítica del modelo previo, las categorías emergentes y el refinamiento de los conceptos analizados en las etapas previas. Dicho modelo fue presentado en un segundo seminario en Santiago al que asistieron 16 expertos (de 31 convocados). Este seminario abarcó un análisis de las etapas anteriores.

### Elaboración de modelo conceptual definitivo

Con base en las críticas efectuadas en el último seminario de expertos, además de la sistematización y organización de la información de las fases anteriores, se generó un MC final. Se compararon las conclusiones de cada etapa y se determinaron el número de AA, sus atributos, relaciones, factores condicionantes y nivel de condicionamiento. Se decide incluir cinco AA y se reorganizan las relaciones con sus condicionantes, de modo de darle posibilidades de comprensión al fenómeno de la alimentación chilena (13). Este modelo fue representado por un diseñador gráfico con ayuda de los investigadores, con el fin de lograr una imagen que facilitara su comunicación y organización conceptual.

## RESULTADOS

**FIGURA 3.**
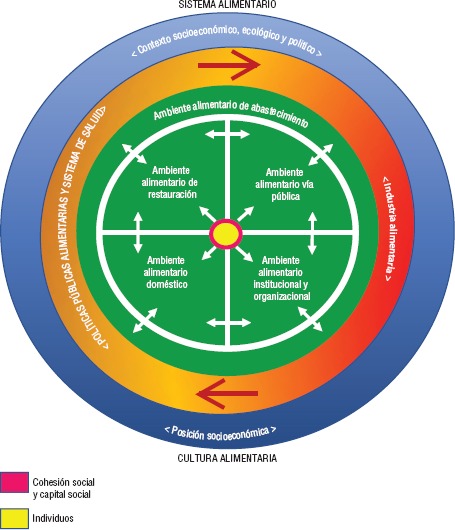
Modelo conceptual sobre ambientes alimentarios y sus condicionantes en Chile

El MC final se muestra en la figura 3. A continuación, se describen cada uno de los componentes del MC.

### Ambientes alimentarios

**FIGURA 4.**
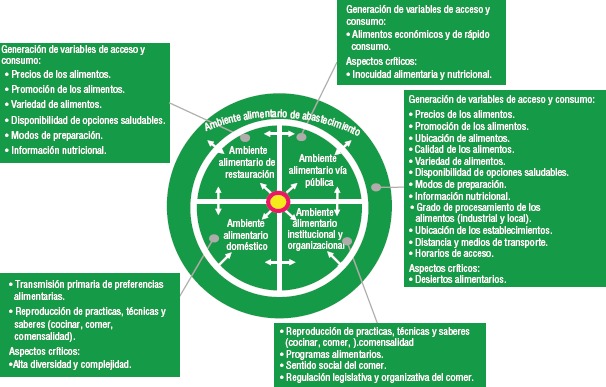
Esquema sobre las funciones relevantes de los ambientes alimentarios.

El modelo incorpora cinco AA que poseen características distintivas (figura 4) y que son entendidos como “determinantes intermedios de la alimentación.” Cada uno actúa de manera independiente, interactúa con otros e influye en la dieta de los individuos.

### Ambiente alimentario doméstico.

El AA doméstico ha sido descrito como uno de los ambientes más complejo (11), debido a la diversidad de hogares y su nivel de implementación para poder producir o transformar los alimentos. Constituye el principal espacio de socialización primaria donde se definen, simbolizan, transmiten y reproducen gran parte de las preferencias y tradiciones alimentarias (18, 19). Los métodos de preparación de los alimentos influyen también en la calidad de la alimentación al interior de este AA (20, 21). Un tema destacado en este ambiente ha sido la pérdida de saberes y tradiciones culinarias (21), históricamente vinculados a los roles femeninos. La salida de la mujer del hogar para insertarse en el mundo asalariado aparece como un tópico recurrente para explicar la pérdida de control familiar sobre el consumo de los alimentos. La pérdida de estos saberes se vincula a una pérdida de soberanía, en la cual la alimentación doméstica es externalizada; esto causa el aumento de la adquisición de alimentos ultraprocesados, caracterizados por poseer una composición nutricional desbalanceada (22). Asimismo, existe una modificación en las prácticas de comensalidad al interior del ambiente doméstico, relacionada con las largas jornadas laborales de los padres o cuidadores (21).

Figura elaborada a partir de datos del presente estudio.

**Ambiente alimentario vía pública.** El AA vía pública se refiere a la venta de alimentos en calles, medios de transporte y otros; se destaca por la presencia de alimentos que pueden ser consumidos de inmediato o que requieren una preparación mínima para el consumo (23). Los precios de venta en este ambiente son bajos, lo que facilita el consumo (23, 24). Por ello, suele reflejar una dimensión popular de la cultura alimentaria local y migrante en las urbes (25).

En este AA, destaca el gran número de vendedores ambulantes y la falta de regulación por parte del Estado del tipo, manipulación y forma de preparación de los alimentos que se expenden, y resaltan los aspectos vinculados con la inocuidad microbiológica y nutricional (cantidad de grasa, azúcar y sal) de los alimentos expendidos (25, 26).

### Ambiente alimentario institucional y organizacional.

Este AA se refiere al lugar donde se venden o proporcionan alimentos a los trabajadores, estudiantes u otros miembros que se desempeñan en instituciones y organizaciones. Incluye las escuelas, universidades, empresas, servicios públicos, hospitales, cárceles y asociaciones de la sociedad civil y sus respectivos casinos y centros de alimentación (cafeterías, quioscos y máquinas expendedoras de alimentos).

Estas definiciones obligan a reconocer el sentido de las reglas y prácticas habituales que poseen las organizaciones, así como los medios temporales, humanos y materiales destinados a lograrlo (27). Estos espacios definen también rituales, tiempos, capacidades, infraestructura y sistemas de regulación sanitaria, mediadas por programas alimentarios y legislación que no son homogéneos en nuestro país, lo que plantea la necesidad de generar estándares nacionales sobre este AA. Es importante no reducir la definición al tipo y cantidad de alimentos accesibles, sino revisar la vigencia y formas de entrega de raciones de los programas alimentarios frente a nuevos gustos y preferencias culturales y socioeconómicas, la organización de los tiempos de alimentación, el rol de los quioscos en las escuelas, el confort durante el tiempo de alimentación escolar y de párvulos y su coherencia con la alimentación del hogar, entre otros.

### Ambiente alimentario de restauración.

Comer fuera del hogar es un hábito que se ha incrementado en todo el mundo en las últimas décadas; incluye comer en restaurantes, locales de comida rápida, bares, hoteles, medios de transportes (aviones, barcos, etc.), además de comer en casas de familiares y amigos (28). Encuestas alimentarias internacionales concluyen que una proporción importante de las calorías que ingiere la población adulta (entre 38% y 74%) provienen de alimentos ingeridos fuera del hogar; esta variación se asocia a la edad, nivel socioeconómico y de ingresos (29).

Los factores que motivan a las personas a comer fuera de su hogar dependen de la edad, género, lugar de residencia (urbano o rural) y nivel socioeconómico. Además, se relacionan con factores culturales (28, 29), los cuales se reflejan principalmente en la selección del tipo de plato o establecimiento; sin embargo, la búsqueda de mayor variedad de alimentos y la conveniencia aparecen como motivaciones comunes en la mayoría de los casos.

### Ambiente alimentario de abastecimiento

El ambiente de abastecimiento posee una doble dimensión. Por una parte, es un AA en sí pero, además, modula las posibilidades de los demás ambientes, loque condiciona la disponibilidad y acceso de alimentos dentro de estos. Esta relación no siempre es unidireccional: al estar mediada por relaciones de mercado, la demanda de un conjunto de alimentos puede modificar la oferta al interior de este AA.

Figura elaborada a partir de datos del presente estudio.

Su principal característica es la distribución espacial de las fuentes de adquisición de alimentos (supermercados, almacenes, ferias, etc.) y su accesibilidad; se considera el tiempo de desplazamiento hacia ellos y los horarios de funcionamiento (30, 31). Un tópico recurrente es la oposición en Chile entre supermercados y ferias libres, marcado por el crecimiento no controlado de los primeros y el estancamiento de las segundas; fenómeno acompañado por una mayor disponibilidad de alimentos ultraprocesados en desmedro de alimentos saludables.

Los factores que condicionan los AA desde el punto de vista estructural son el sistema y la cultura alimentaria, los determinantes sociales, la industria alimentaria, las políticas en alimentación y nutrición y los individuos y la cohesión social.

### Sistema y cultura alimentaria.

El sistema alimentario y la cultura alimentaria son pensados como las categorías más amplias del MC. Son entendidos como determinantes sociales de la salud (y la alimentación), pero cuya relevancia hermenéutica es mayor a otras, que operan indirectamente (32), son dimensiones complementarias e indisociables del mismo fenómeno. Cada elemento del sistema alimentario posee una dimensión cultural propia y que permite significarlo de forma local. Si bien todo el sistema alimentario debe dar cuenta de la producción, distribución, elaboración, consumo y desecho de los alimentos, la cultura alimentaria indica qué se produce, cómo se distribuye, quién lo elabora, cuando se come y qué se desecha, entre otras variables (16). La cultura modula las técnicas de preparación (33) y los tiempos de consumo (34), así como los ritos de comensalidad que simbolizan y refuerzan el orden social (34). Esto hace que exista una compleja mezcla de factores sociales, comunitarios e individuales que condicionan qué, cuándo y dónde se come; operan de manera directa desde el sistema alimentario y de manera indirecta mediante las vías políticas, económicas, sociales y culturales que generan estratificación social e influencian la vida y calidad de vida de la población (32).

### Determinantes sociales de los AA y alimentación.

Este aspecto remite a la dimensión macroestructural de la sociedad nacional, tanto actual como pasada; también a las condiciones materiales que estructuran el sistema alimentario y cómo esto influye a los AA. Constituyen los factores estructurales e intermedios que condicionan la forma en que la sociedad accede, interactúa y evoluciona con los AA, y su conexión con el sistema alimentario. Se asume que las instituciones ayudan a distribuir y regular el poder de acceso a bienes y servicios y, por lo tanto, cómo se accede a calidades y cantidades de alimentos según el nivel socioeconómico y el territorio administrativo y geográfico en el cual se vive.

El modelo de los determinantes sociales de la salud (DSS) (15) ayuda a distinguir elementos estructurales que condicionan a los AA; se considera que estos últimos son “determinantes intermedios” de la alimentación. El enfoque permite comprender cómo países con problemas y trayectorias epidemiológicas aparentemente similares poseen historias de determinación social diversas (35). Además, permite comprender cómo el modelo ideológico y económico dominante influye en el mercado laboral y la forma en que la población concibe el consumo, los bienes y servicios básicos, y su influencia sobre el mercado alimentario (35). Así, los DSS afectan los estilos de vida, y contribuyen a la generación de desigualdad en alimentación. También se mencionan otros factores que estructuran los AA como el contexto socioeconómico y político, cultura y valores sociales, clase socioeconómica, educación, género, etnicidad, etapa generacional, nivel de ingreso, entre otros.

### Industria alimentaria.

Se define como un sector de la producción industrial que se encarga de la transformación y la conservación de materias primas que se utilizan para el consumo alimentario (36). Es uno de los principales agentes que modelan la salud de la población y un elemento crítico que estructura los AA a nivel mundial (37). Cabe destacar la importancia que la industria alimentaria tiene para la economía, ya que genera grandes entradas de dinero para el presupuesto del país (38). Si bien la industria alimentaria es uno de los mayores contribuyentes de alimentos disponibles en cada AA, recibe críticas por la responsabilidad que posee en la generación de AA poco saludables, asociado a la incidencia de compañías transnacionales de alimentos con la alta prevalencia de enfermedades no transmisibles asociadas a la nutrición (39). Se destaca como crítica a la industria alimentaria, el uso del mercadeo para promocionar alimentos no saludables, en especial dirigido a menores de edad.

### Políticas en alimentación y nutrición.

Las políticas alimentarias son un conjunto de acciones cuyo objetivo es mejorar la dieta de la población (40). Conforme aumentan los problemas asociados a la adopción de dietas no saludables, aumenta el diseño de políticas orientadas a promover una alimentación saludable (41).

Así, los gobiernos están habilitados para emprender acciones que permitan tanto afrontar los problemas del mercado alimentario como para ofrecer alimentos saludables, y pueden regular la oferta de alimentos relacionados con el problema de la obesidad (42). Sin embargo, esto último se ve entorpecido por el creciente interés comercial con que se desarrollan las políticas públicas (43). Algunas políticas alimentarias han sido criticadas por perjudicar la economía, sobre todo a los pequeños comerciantes que venden alimentos ultraprocesados (41). Los grupos consultados indicaron que los gobiernos no han emprendido suficientes acciones para controlar los sistemas alimentarios y son necesarios más estudios para fortalecer esta área.

### Individuos y cohesión social.

Aun cuando este MC prioriza el estudio de la CA desde los AA, no podemos dejar de referirnos a los individuos, dado que son quienes, en última instancia, realizan las conductas y transitan por los AA.

Aunque las decisiones que hacen los sujetos sobre los alimentos involucran una serie de variables individuales, estas se pueden cumplir o no según lo que tienen disponible en sus hogares (ambiente doméstico), vecindarios (ambiente de abastecimiento, espacio público o comida elaborada) y lugares de trabajos o escuelas (ambiente organizacional).

El capital y la cohesión sociales son factores que median las desigualdades más estructurales del sistema alimentario y social. El capital social está compuesto por todos los recursos potenciales o actuales que se relacionan con una red de soporte social de conocimiento y reconocimiento mutuos (44). Desde una dimensión alimentaria, el capital social puede articularse de múltiples maneras: mediante la formación de redes de abastecimiento y elaboración (ollas comunes) y también mediante grupos de apoyo que transmiten saberes para mejorar la alimentación de la comunidad.

## DISCUSIÓN

En el presente estudio se utilizó una revisión bibliográfica y técnicas cualitativas participativas para elaborar un MC que permitiera explorar cómo son condicionados los AA. El modelo presenta cinco AA, entendidos en especial desde su dimensión física (como espacios particulares), pero comprendiendo que se encuentran atravesados por dimensiones culturales y sociales (las cuales tienen una gran influencia en la mayoría de los países sobre las preferencias y práctica alimentarias de las personas. Es así que estos ambientes alimentarios condicionarían tanto el consumo, como la selección y preparación de los alimentos (dimensión que incluye el uso de artefactos y funciones), considerando dimensiones ideológicas, económicas, entre otras.

El presente MC sugiere que los AA son entornos que los individuos y colectivos utilizan para producir, comprar, almacenar, preparar, comer y desechar alimentos en distintas formas y formatos. Estos entornos están condicionados de manera indirecta por factores estructurales multinivel, los cuales operan a nivel de la gran mayoría de los países y son reconocidos por organismos internacionales como la OMS y FAO (sistema alimentario, cultura alimentaria, contexto socioeconómico, ecológico y político, posición social, industria alimentaria, políticas públicas alimentarias y sistema de salud). Estos condicionantes de los AA están relacionados entre sí a través de rutinas derivadas del estilo de vida de los individuos y colectivos que viven en un territorio geográfico y administrativo común.

A nivel internacional, existe una vasta literatura sobre modelos teóricos conceptuales que contribuyen a entender la CA en forma directa y revisan, de manera indirecta o inespecífica los AA (por ejemplo, el modelo socioecológico) (45). Otros modelos existentes describen las influencias de AA sobre la dieta, sin tener en cuenta los factores que los condicionan (19, 46, 47). Otro grupo de estudios se orientan al análisis de un AA específico, sin integración con otros ambientes (48–50). A la fecha, existen pocos modelos que tratan de explicar e integrar la complejidad de los AA y sus condicionantes mediante la incorporación de temáticas como los sistemas alimentarios o industria alimentaria. Uno de estos modelos es el generado por Glanz et al. en el 2005 (14), que ha sido utilizado para la generación de otros estudios (49), y que fue incorporado como base en el presente trabajo.

Resulta interesante situar este estudio conceptual y su metodología de desarrollo dentro del debate de la generación de índices de vigilancia de ambientes que se da hoy en día a nivel internacional debido a que, de manera simultánea, se intenta generar indicadores globales, aunque con pertinencia local. Este estudio es concordante con gran parte de los dominios que revela la Red internacional para la investigación, monitoreo y apoyo a la acción para la alimentación, obesidad y enfermedades no transmisibles (INFORMAS por sus siglas en inglés) y su herramienta Food-EPI (9, 10); sin embargo se debe prestar especial cuidado a la forma en que estos dominios se conciben, se expresan y se representan a nivel local. Mientras para INFORMAS los AA son “entornos físicos, económicos, políticos y socioculturales, oportunidades y condiciones que influencian las elecciones de alimentos, bebidas y estado nutricional”, el modelo propuesto mueve los límites de las elecciones a todolo que sucede en ellos desde la producción de alimentos hasta su desecho y eliminación. Por otro lado, mientras en nuestra definición se permite problematizar las acciones o monitoreo en entornos específicos que son permeables a las rutinas diarias de una comunidad, los indicadores derivados de la definición de INFORMAS sitúan la mayoría de sus indicadores a niveles de microsistema de regulaciones, pero que no permiten ver con claridad, como la clase social, la educación, el género y el acceso a la salud podrían ser, a su vez, determinantes intermedios de dichos entornos. Este artículo propone el seguimiento académico de los conceptos que se utilizan, la coherencia entre estos y los indicadores utilizados para la toma razonable de decisiones en materia de políticas públicas relacionadas con problemas persistentes que atañen a la dieta, el ejercicio y la obesidad.

Si bien el presente trabajo presenta información de utilidad para el estudio de AA, es importante mencionar sus limitaciones. Primero, la revisión de la literatura no siguió criterios específicos señalados por organismos internacionales como PRISMA o PROSPERO. Segundo, el número de participantes, tanto a los seminarios de expertos como a los diálogos ciudadanos, fue menor a lo esperado, lo que puede limitar la discusión sobre otros aspectos de importancia.

El presente estudio propone un modelo conceptual para el estudio de AA y sus condicionantes. El modelo representa un conjunto de definiciones, conceptos y relaciones, que interactúan en forma multidireccional de manera compleja, y que creemos ayudan a comprender de forma integral cómo son condicionados los AA o la conducta alimentaria. En este sentido, este trabajo invita a formular preguntas sobre la práctica alimentaria y las debilidades que tienen ciertas políticas orientadas a un solo sector (por ejemplo, el de la salud) y relevar la necesidad de un trabajo multidisciplinar e intersectorial en el estudio y la generación de estrategias orientadas a mejorar los AA, considerando la heterogeneidad del país.

### Agradecimientos.

Los autores agradecen la asesoría brindada por el Departamento de Alimentos y Nutrición, perteneciente a la División de Políticas Públicas Saludables y Promoción, del Ministerio de Salud de Chile. Además, se agradece a los expertos y miembros de la ciudadanía quienes participaron de manera voluntaria en la discusión de los modelos preliminares. Por último, agradecemos a Pedro Masfarrer por el diseño gráfico del modelo.

### Financiamiento.

Esta investigación fue financiada por la Subsecretaría de Salud Pública del Ministerio de Salud de Chile, en su licitación número 757-230-LE15.

### Declaración

Las opiniones expresadas en este manuscrito son responsabilidad del autor y no reflejan necesariamente los criterios ni la política de la RPSP/PAJPH y/o de la OPS
